# Comparison of Ion Balance and Nitrogen Metabolism in Old and Young Leaves of Alkali-Stressed Rice Plants

**DOI:** 10.1371/journal.pone.0037817

**Published:** 2012-05-24

**Authors:** Huan Wang, Zhihai Wu, Jiayu Han, Wei Zheng, Chunwu Yang

**Affiliations:** 1 Key laboratory of Molecular Epigenetics of MOE, Northeast Normal University, Changchun, Jilin Province, China; 2 Department of Agronomy, Jilin Agricultural University, Changchun, Jilin Province, China; 3 Northeast Institute of Geography and Agroecology, Chinese Academy of Sciences, Changchun, China; United States Department of Agriculture, United States of America

## Abstract

**Background:**

Alkali stress is an important agricultural contaminant and has complex effects on plant metabolism. The aim of this study was to investigate whether the alkali stress has different effects on the growth, ion balance, and nitrogen metabolism in old and young leaves of rice plants, and to compare functions of both organs in alkali tolerance.

**Methodology/Principal Findings:**

The results showed that alkali stress only produced a small effect on the growth of young leaves, whereas strongly damaged old leaves. Rice protected young leaves from ion harm via the large accumulation of Na^+^ and Cl^−^ in old leaves. The up-regulation of *OsHKT1;1*, *OsAKT1*, *OsHAK1*, *OsHAK7*, *OsHAK10* and *OsHAK16* may contribute to the larger accumulation of Na^+^ in old leaves under alkali stress. Alkali stress mightily reduced the NO_3_^−^ contents in both organs. As old leaf cells have larger vacuole, under alkali stress these scarce NO_3_^−^ was principally stored in old leaves. Accordingly, the expression of *OsNRT1;1* and *OsNRT1;2* in old leaves was up-regulated by alkali stress, revealing that the two genes might contribute to the accumulation of NO_3_^−^ in old leaves. NO_3_^−^ deficiency in young leaves under alkali stress might induce the reduction in *OsNR1* expression and the subsequent lacking of NH_4_^+^, which might be main reason for the larger down-regulation of *OsFd-GOGAT* and *OsGS2* in young leaves.

**Conclusions/Significance:**

Our results strongly indicated that, during adaptation of rice to alkali stress, young and old leaves have distinct mechanisms of ion balance and nitrogen metabolism regulation. We propose that the comparative studies of young and old tissues may be important for abiotic stress tolerance research.

## Introduction

Alkali stress is an important agricultural contaminant and has complex effects on plant metabolism. There are 831 million hectares of soil in the world that are affected by salt stress. Of this area, alkalinized soils underlie 434 million hectares, while saline soils underlie 397 million hectares [1]. Soil alkalization causes severe problems in some areas. For example, more than 70% of land area in northeast China is alkaline grassland [2], sometimes with a soil pH>10 [3]. Plants on these soils endure concomitant salt and alkali (high-pH) stresses, making it difficult to grow, and only a few species of plants can survive on such soils. Some reports have clearly demonstrated that alkaline salt stress (NaHCO_3_ and/or Na_2_CO_3_) and neutral salt stress (NaCl and/or Na_2_SO_4_) are two distinct types of stress that affect plants and that they should be referred to as alkali stress and salt stress, respectively [4]. In fact, alkali stress has been shown to cause much stronger destructive effects on plants than salt stress [5], [6], [7]. However, to date, salt stress studies have generally emphasized NaCl [8], while little attention has been given to alkali stress.

It is well known that salt stress has different effects on old and young tissues [8]. For example, salt stress produces distinct effects on the growth, compatible solutes accumulation and ion metabolism of old and young leaves [9], [10], [11], [12], [13], [14]. Salt stress in a soil generally involves osmotic stress and ion-induced injury [15]. Comparison of alkali stress with salt stress reveals an added high-pH effect of alkali stress. The high-pH environment surrounding the roots can cause metal ions and phosphorus to precipitate, and the loss of the normal physiological functions of the roots and the destruction of root cell structure [5], [6], [7]. Alkali stress can inhibit the absorption of inorganic anions such as Cl^−^, NO_3_^−^ and H_2_PO_4_^−^, greatly affects the selective absorption of K^+^–Na^+^, and breaks the ionic balance in tissue [6], [16]. Although several attentions have been given to alkali stress [1], [5], [6], [7], [16], [17], [18], the comparative study of old and young tissues was lacking. Old and young tissues may play different roles in alkali tolerance as in salt tolerance. Thus, the understanding of comparative effects of alkali stress on old and young tissues may be important for alkali tolerance research.

The maintenance of K^+^ and Na^+^ homeostasis is crucial for alkali tolerance [6], [16]. Many transporters of K^+^ and Na^+^ have been identified to date. In leaves of plants, salt overly sensitive (SOS) salt tolerance pathway may play important roles in Na^+^ eduction [8], [19]. In addition, some members of the high affinity K^+^ transporter (HKT) family, such as OsHKT1; 5 and AtHKT1;1, mediate Na^+^ exclusion from leaves via Na^+^ removal from the xylem sap [8]. Our previous studies showed that alkali stress may strongly affect assimilation and/or uptake of nitrate in rice [20] and other plants [6], [16]. Thus, nitrogen metabolism regulation may be important for rice alkali tolerance. NO_3_^−^ is reduced to nitrite by nitrate reductase (NR) and then to NH_4_^+^ by nitrite reductase (NiR). NH_4_^+^ is incorporated into organic molecules by glutamine synthetase (GS) and glutamate synthase (GOGAT) or alternative glutamate dehydrogenase (GDH) pathway [21]. Glutamine synthetase (GS) primarily exists as two isozymes with different subcellular localisations: GS1 in the cytosol and GS2 in chloroplasts/plastids. In rice, *OsGS1;1*, *OsGS1;2* and *OsGS1;3*, encode GS1. *OsGS1;1* and *OsGS1;2* are especially abundant in the aerial parts and roots, respectively, whereas *OsGS1;3* is present only in the spikelets [22].

In this study, we chose rice plants as the experimental material. The study was designed to investigate whether alkali stress has different effects on the growth, ion balance, and nitrogen metabolism in the old and young leaves of rice, and to compare functions of both organs in rice alkali tolerance.

## Methods

### Plant Growth Conditions

Tong-35, a major rice cultivar in north China, was chosen as the test organism. Seeds were germinated and grown in petri dishes for 6 d in a growth cabinet (30°C during the day and 25°C during the night, 16/8 h photoperiod at 250 µmol m^−2^ s^−1^). Seedlings were then transferred to buckets containing 2000 mL of sterile nutrient solution for solution culture. The nutrient solution was replaced daily. The buckets were placed in a growth chamber that was maintained at 27.0±1.5°C during the day and 22.0±1.5°C during the night, under a 16/8 h photoperiod at 250 µmol m^−2^ s^−1^. The nutrient solution used in this work accorded to the components described by the International Rice Research Institute [23], and contained 1.44 mM NH_4_NO_3_, 0.32 mM NaH_2_PO_4_, 0.6 mM K_2_SO_4_, 1.0 mM CaCl_2_, 1.6 mM MgSO_4_, 0.072 mM Fe-EDTA, 0.2 mM Na_2_SiO_3_, 9.1 µM MnCl_2_, 0.154 µM ZnSO_4_, 0.156 µM CuSO_4_, 18.5 µM H_3_BO_3_ and 0.526 µM H_2_MoO_4_ at pH 5.2.

### Stress Treatment

Two alkaline salts (NaHCO_3_ and Na_2_CO_3_) were selected based on the salt components and pH in the majority of alkaline soils in northeast China. Two alkaline salts were mixed in a 9∶1 molar ratio (NaHCO_3_∶Na_2_CO_3_) as the alkali stress treatment. The total salt concentration was set at 50 mM (pH 9.10). After 22 days of growth in hydroponic medium, rice plants were subjected to alkali stress by transferring them to another bucket containing 2000 mL of the treatment solution amended with the above nutrients and 50 mM stress salts. A bucket including 20 seedlings represented one replicate, and there were four replicates per treatment. 8 buckets of seedlings were randomly divided into 2 sets, four buckets per set. Each bucket was considered as one replicate with four replicates per set, one set was used as control, and another set was treated with alkali stress. Namely, the experiment has four biological replicates. Treatment solutions were replaced daily. The nutrient solution without stress salts was used as a control. The 20 seedlings in each bucket were harvested after treatment for 6 d.

### Measurements of Physiological Indices

Membrane permeability can be reflected by the electrolyte leakage rate, which was determined with the ameliorated method of Lutts et al. (1996) [24]. One fresh whole leaf from each bucket was washed three times with deionized water to remove surface adhered electrolytes, then was placed in a closed cuvette containing 20 mL of deionized water at 25°C for 5 h. The electrical conductivity of the solution (EC1) was determined with a conductivity gauge. After this the cuvette was autoclaved at 100°C for 20 min, and the electrical conductivity of the solution (EC2) was determined. Electrolyte leakage rate can be defined as follows: Electrolyte leakage rate (%) = (EC1/EC2)×100.

The young and old leaves of 10 seedlings in each bucket were separated and mixed, then immediately frozen in liquid nitrogen and then stored at −70°C for RNA isolation and the measurements of pigments. Carotenoids, chlorophyll a and chlorophyll b were determined with the method of Zhu (1993) [25], and expressed in mg g^−1^ FW. Another 10 seedlings in each bucket were washed with distilled water, after which the old (second leaf at bottom) and young leaves were separated and freeze-dried. Then the dry samples of plant material were levigated and mixed for physiological index measurements. Dry samples of plant material (50 mg) were treated with 10 mL deionized water at 100°C for 2 h, and the extract used to determine the contents of free inorganic ions and organic acids (OAs). The contents of NO_3_^−^, Cl^−^, H_2_PO_4_^−^, SO_4_^2−^ and oxalic acid were determined by ion chromatography (DX-300 ion chromatographic system; AS4A-SC ion-exchange column, CD M-II electrical conductivity detector, mobile phase: Na_2_CO_3_/NaHCO_3_ = 1.7/1.8 mM; DIONEX, Sunnyvale, USA). Other OAs were also determined by ion chromatography (DX-300 ion chromatographic system; ICE-AS6 ion-exclusion column, CDM-II electrical conductivity detector, AMMS-ICE II suppressor, mobile phase: 0.4 mM heptafluorobutyric acid; DIONEX, Sunnyvale, USA). A flame photometer was used to determine K^+^ and Na^+^ contents. Ammoniacal nitrogen and soluble sugars were measured, respectively, using ninhydrin and anthrone methods [26].

### Quantitative Real Time PCR Analysis

We extracted the total RNA from the young and old leaves of seedlings grown under stress or non-stress conditions using TRIzol reagent (Invitrogen). The RNA was treated with DNaseI (Invitrogen), reverse-transcribed using SuperScriptTM RNase H-Reverse Transcriptase (Invitrogen), and then subjected to real time PCR analysis using gene-specific primers. The functions and sequence informations of the genes used in this study had been reported. The gene-specific primers and corresponding references are listed in Table S1 online. PCR amplification was conducted with an initial step at 95°C for 1 min followed by 40 cycles of 5 s at 95°C, 10 s at 60°C and 30 s at 72°C. Amplification of the target gene was monitored every cycle by SYBR Green. Amplification of the rice *UBQ5* (GenBank Accession AK061988) mRNA was used as an internal quantitative control [27], [28], [29]. The relative expression of the target genes was calculated using the ΔCt method [30]. We optimized PCR reaction system, after which the amplification efficiencies of each target gene and reference gene were approximately equal.

### Statistical Analysis

Statistical analysis of the data was performed using the statistical program SPSS 13.0 (SPSS, Chicago, USA). All data were represented by an average of the four biological replicates and the standard errors (S.E.). Statistically significant between old and young leaves at same stress condition was determined by *t* test.

## Results

### Growth and Ion Accumulation

**Figure 1:**
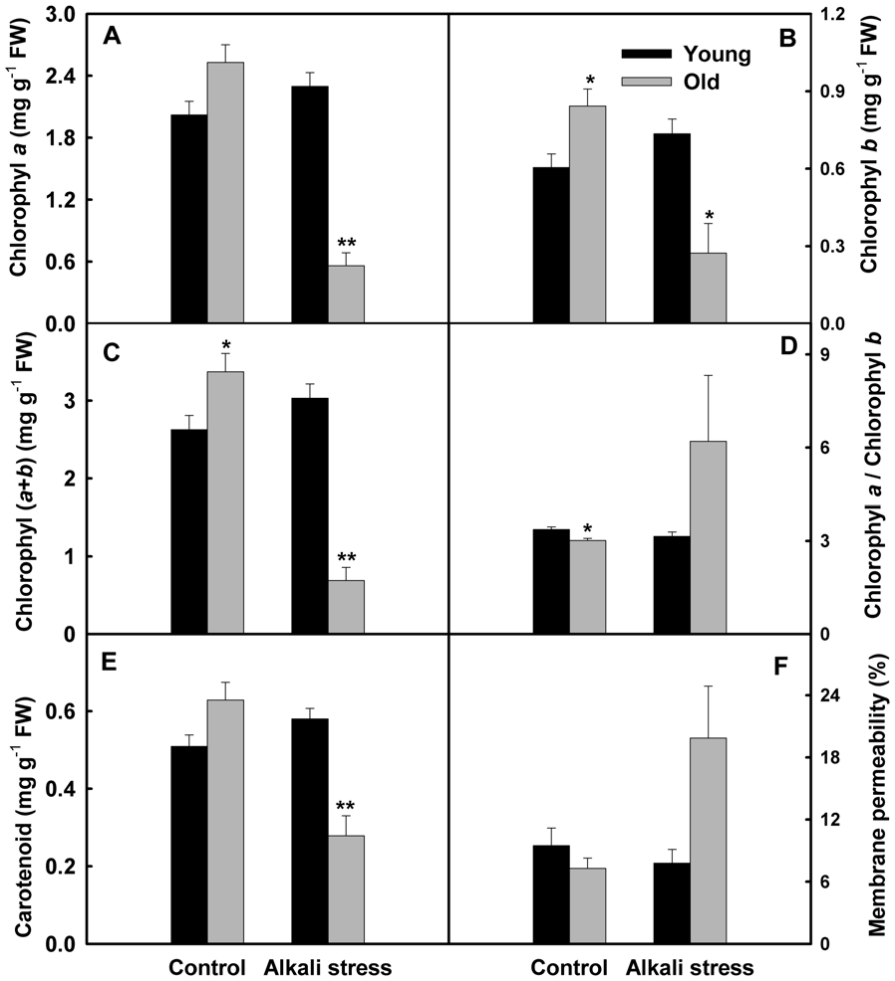
Effects of alkali stress on the growth in young and old leaves of rice seedlings. The values are means (± SE) of four biological replicates. Statistically significant between organs at same stress condition was determined by *t*-test, and marked as * (*P*<0.05) and ** (*P*<0.01). The seedlings were subjected to 50 mM alkali stress (NaHCO_3_∶Na_2_CO_3_ = 9∶1; pH 9.10) stresses for 6d.

**Figure 2:**
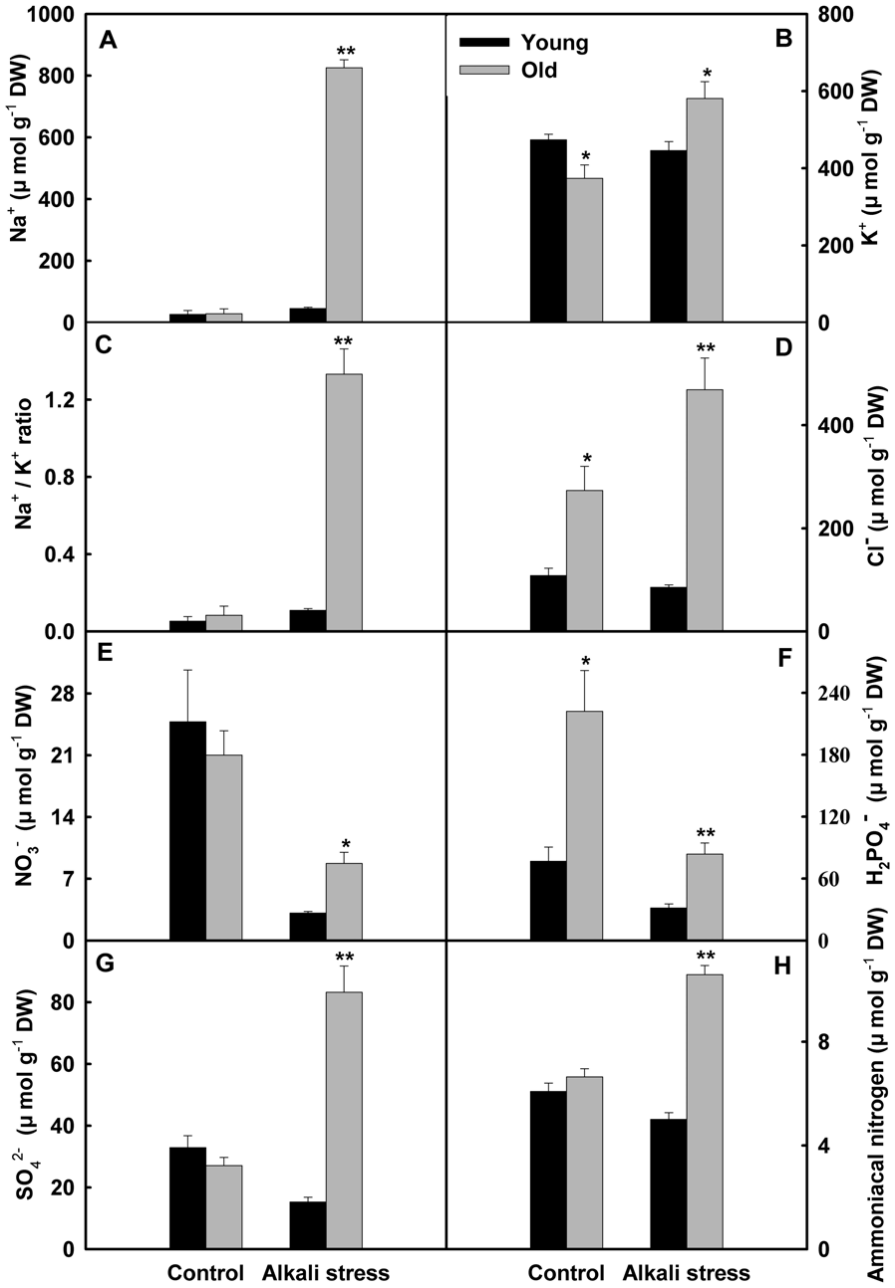
Effects of alkali stress on the contents of inorganic ions in young and old leaves of rice seedlings. The values are means (± SE) of four biological replicates, and each replicate consisted of a pool of 10 plants. Statistically significant between organs at same stress condition was determined by *t*-test, and marked as * (*P*<0.05) and ** (*P*<0.01). The seedlings were subjected to 50 mM alkali stress (NaHCO_3_∶Na_2_CO_3_ = 9∶1; pH 9.10) stresses for 6d.

**Figure 3:**
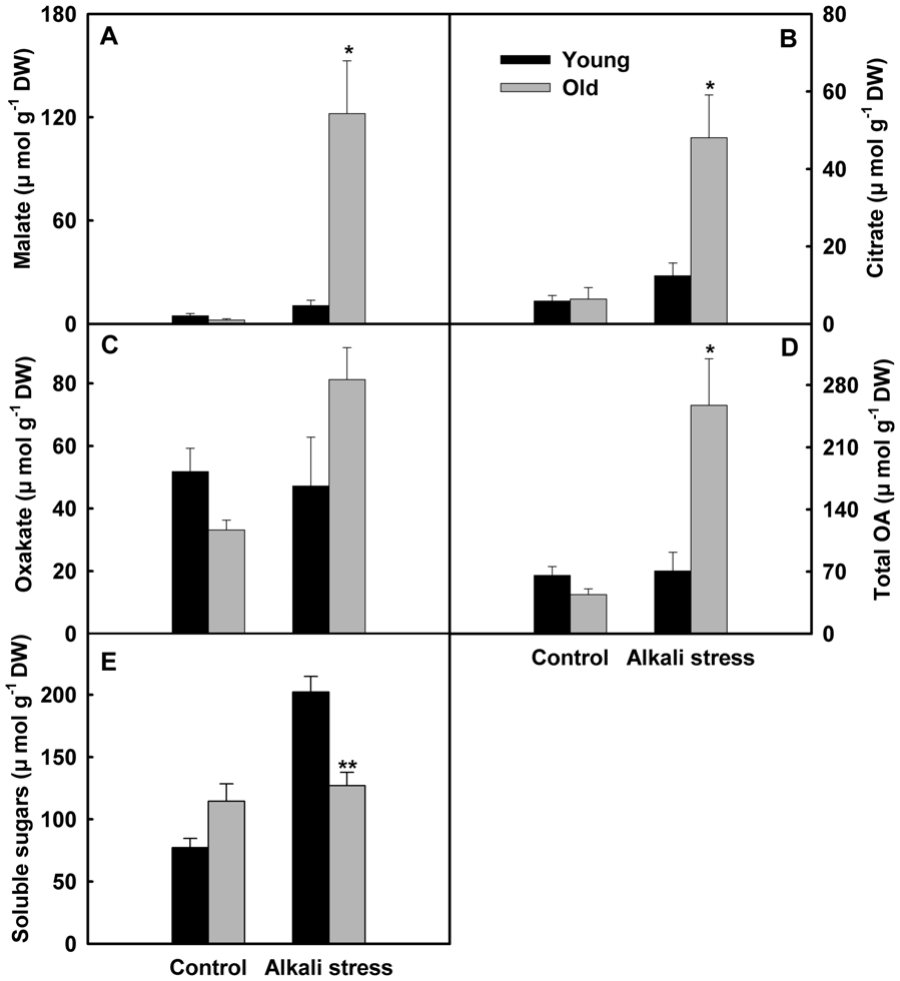
Effects of alkali stress on the contents of organic acids (OA) and soluble sugars in young and old leaves of rice seedlings. The values are means (± SE) of four biological replicates, and each replicate consisted of a pool of 10 plants. Statistically significant between organs at same stress condition was determined by *t*-test, and marked as * (*P*<0.05) and ** (*P*<0.01). The seedlings were subjected to 50 mM alkali stress (NaHCO_3_∶Na_2_CO_3_ = 9∶1; pH 9.10) stresses for 6d.

Alkali stress showed a stronger inhibition effect on the growth of old leaves than that of young leaves (Fig. 1). Alkali stress only has small effect on young leaves, whereas mightily increased electrolyte leakage rate and decreased the contents of carotenoids, chlorophyll a and chlorophyll b (Fig. 1). In addition, alkali stress clearly changed chlorophyll a/chlorophyll b ratio in old leaves. The effects of alkali stress on Na^+^, K^+^, Na^+^/K^+^ ratio, Cl^−^, SO_4_^2−^, ammoniacal nitrogen, and organic acids in young leaves were no significant, but alkali stress strongly stimulated their accumulation in old leaves (Figs. 2 and 3). Moreover, malate, citrate and oxalate were the dominant components of both young and old leaves under alkali stress, while only trace amounts of succinate, acetate, formate and lactate were detected. Thus, we only listed the results of malate, citrate and oxalate in Fig. 3. Alkali stress reduced the NO_3_^−^ contents in both old and young leaves, with reduction in young leaves greater than in old leaves. Under alkali stress, the NO_3_^−^ content in old leaves was much higher than that in young leaves (Fig. 2E). Alkali stress decreased the H_2_PO_4_^−^ contents in both old and young leaves (Fig. 2F). Alkali stress elevated the soluble sugars content in young leaves but did not influence its content in old leaves (Fig. 3E).

### Ion Metabolism

**Figure 4:**
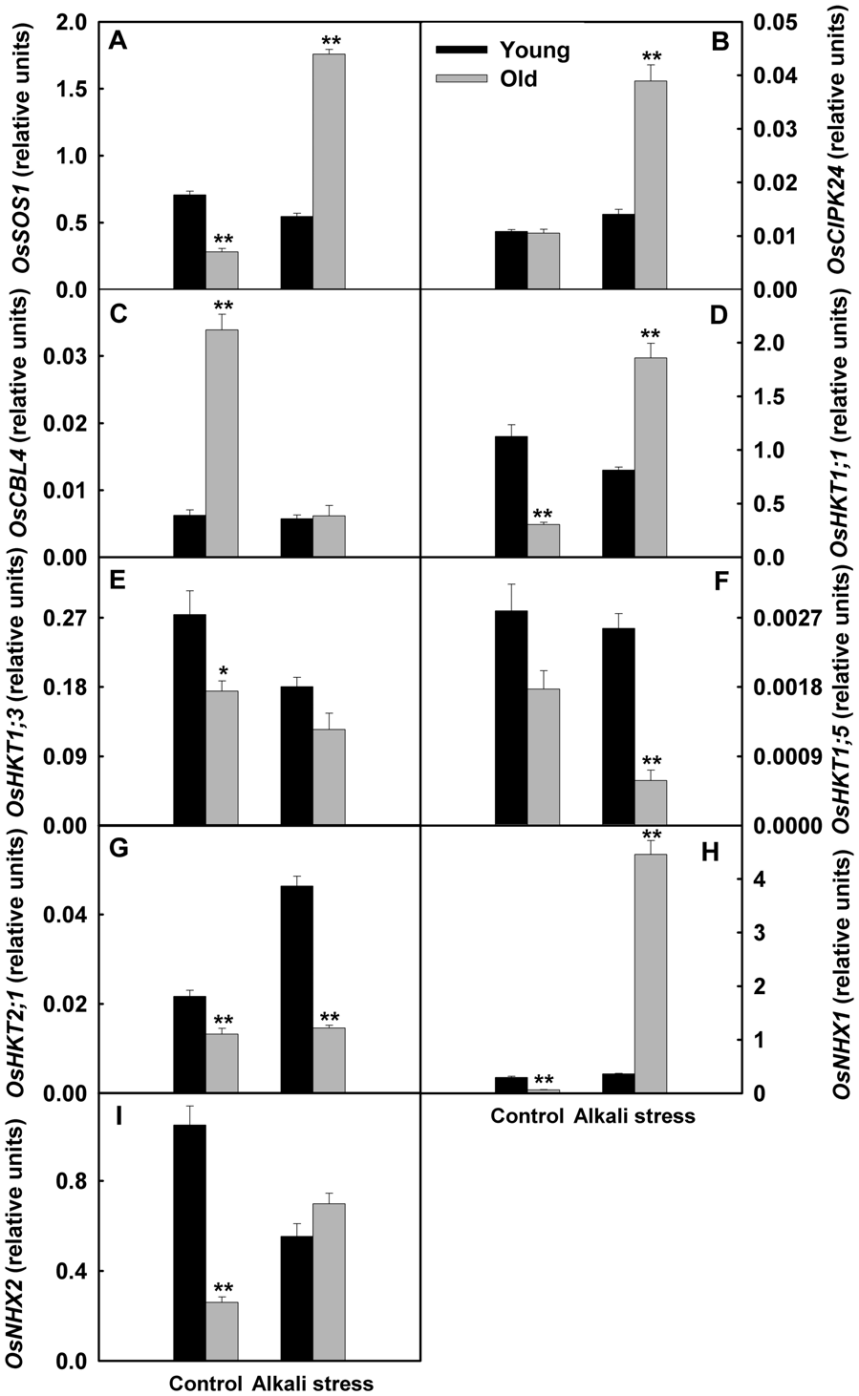
Effects of alkali stress on the expression of *OsSOS* pathway genes and *OsHKT* gene family in young and old leaves of rice seedlings. The values are means (± SE) of four biological replicates, and each replicate consisted of a pool of 10 plants. Statistically significant between organs at same stress condition was determined by *t*-test, and marked as * (*P*<0.05) and ** (*P*<0.01). The seedlings were subjected to 50 mM alkali stress (NaHCO_3_∶Na_2_CO_3_ = 9∶1; pH 9.10) stresses for 6d.

**Figure 5:**
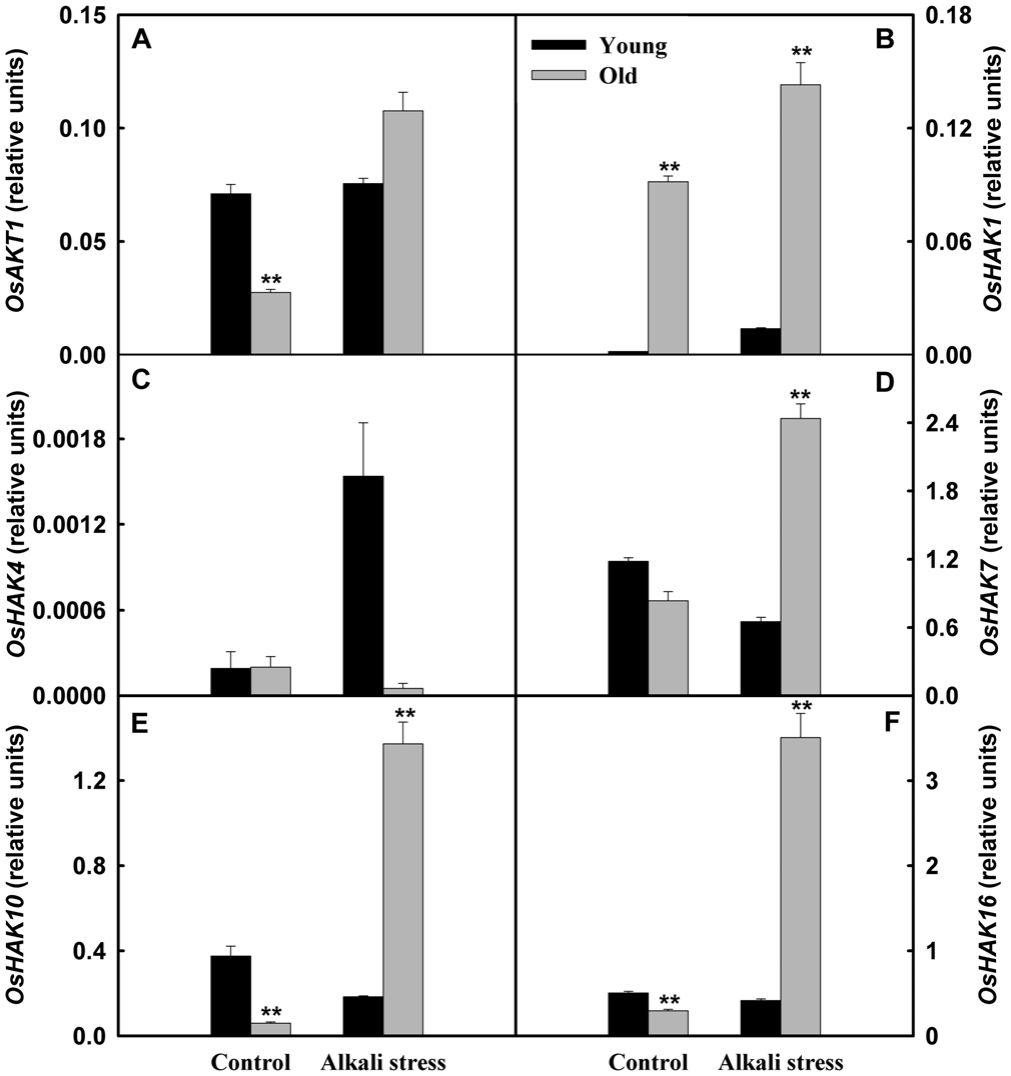
Effects of alkali stress on the expression of *OsAKT1* and *OsHAK* gene family in young and old leaves of rice seedlings. The values are means (± SE) of four biological replicates, and each replicate consisted of a pool of 10 plants. Statistically significant between organs at same stress condition was determined by *t*-test, and marked as * (*P*<0.05) and ** (*P*<0.01). The seedlings were subjected to 50 mM alkali stress (NaHCO_3_∶Na_2_CO_3_ = 9∶1; pH 9.10) stresses for 6d.

The responses of the genes related to K^+^/Na^+^ metabolism to alkali stress were diverse. The expression levels of several genes, such as *OsSOS1*, *calcineurin B-like interacting protein kinase 24 (OsCIPK24)*, *Na^+^/H^+^ exchanger 1* (*OsNHX1*), *OsNHX2*, *OsHKT1;1*, *low affinity K^+^ transporter 1* (*OsAKT1*), *KUP/HAK/KT K^+^ transporter 1* (*OsHAK1*), *OsHAK7*, *OsHAK10*, and *OsHAK16*, in old leaves were clearly up-regulated by alkali stress, but their expression levels in young leaves were not affected (Figs. 4 and 5). Alkali stress only produced small effects on expression of *calcineurin B-like 4 (OsCBL4)* and *OsHKT1;5* in young leaves, but reduced their expression levels in old leaves. Interestingly, alkali stress did not influence *OsHAK4* expression in old leaves, whereas strongly stimulated its expression in young leaves (Fig. 5C). The expression levels of *OsHKT1;3* in both organs were reduced by alkali stress (Fig. 4E).

### NH_4_^+^ Assimilation

**Figure 6:**
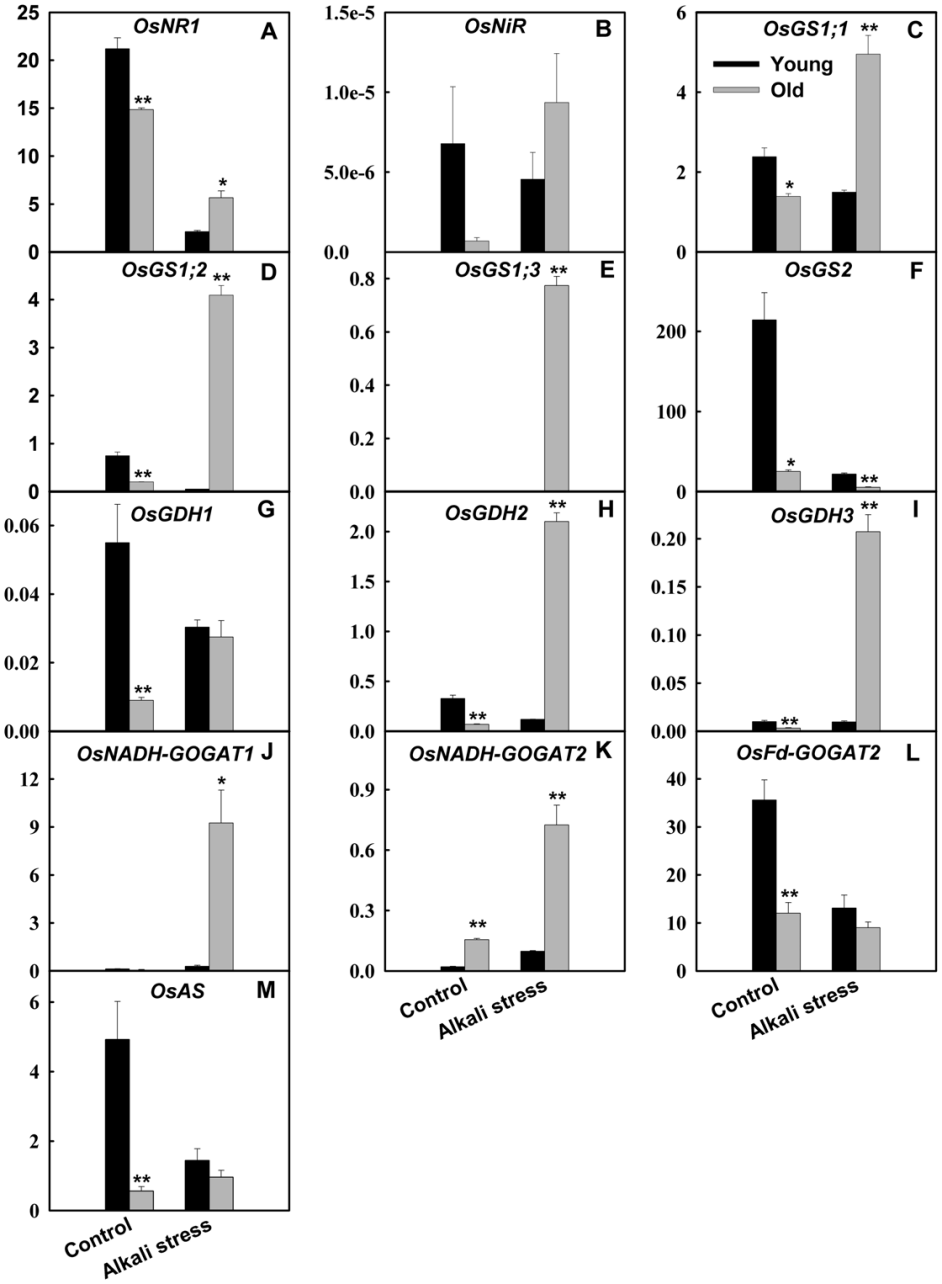
Effects of alkali stress on the expression (relative units) of genes involved in NH_4_^+^ assimilation in young and old leaves of rice seedlings. The values are means (± SE) of four biological replicates, and each replicate consisted of a pool of 10 plants. Statistically significant between organs at same stress condition was determined by *t*-test, and marked as * (*P*<0.05) and ** (*P*<0.01). The seedlings were subjected to 50 mM alkali stress (NaHCO_3_∶Na_2_CO_3_ = 9∶1; pH 9.10) stresses for 6d.

*OsGS2* was dominant *OsGs* family member in leaves, and its expression level in leaves was much higher than other members (Fig. 6). Similarity, *OsFd-GOGAT* was dominant *OsGOGAT* family member in leaves. Alkali stress only has small effects on the expression of *OsFd-GOGAT* and *OsGS2* in old leaves, and mightily reduced their expression in young leaves. Alkali stress only produced small effects on expression of *OsGS1 family*, *OsNADH-GOGAT1*, *OsNADH-GOGAT2*, *OsGDH2*, and *OsGDH3* in young leaves, and strongly stimulated their expression in old leaves (Fig. 6). Alkali stress increased the expression level of *OsGDH1* in old leaves, whereas decreased its expression in young leaves. Alkali stress down-regulated the expression of *asparagine synthetase* (*OsAS*) in young leaves (Fig. 6M), did not influence its expression in old leaves. Alkali stress down-regulated the *OsNR1* expression in both tissues, with reduction in young leaves greater than in old leaves (Fig. 6A). Alkali stress reduced the expression of *OsNiR* in young leaves, and increased its expression in old leaves.

### Nitrogen Uptake

**Figure 7:**
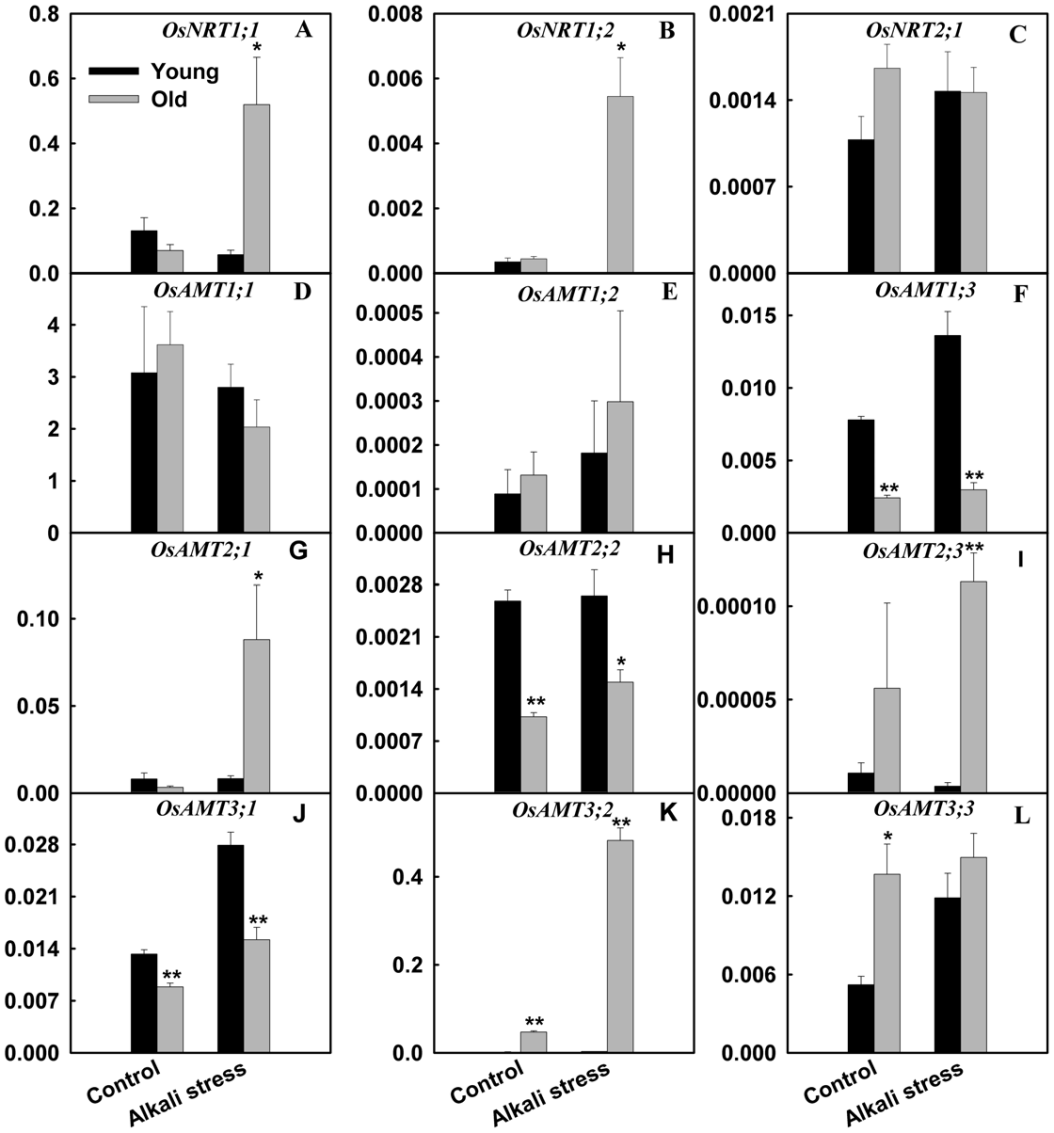
Effects of alkali stress on the expression (relative units) of *OsAMT* and *OsNRT* gene families in young and old leaves of rice seedlings. The values are means (± SE) of four biological replicates, and each replicate consisted of a pool of 10 plants. Statistically significant between organs at same stress condition was determined by *t*-test, and marked as * (*P*<0.05) and ** (*P*<0.01). The seedlings were subjected to 50 mM alkali stress (NaHCO_3_∶Na_2_CO_3_ = 9∶1; pH 9.10) stresses for 6d.

The expression levels of nitrate transporter *1;1* (*OsNRT1;1*) and O*sNRT1;2* in old leaves were up-regulated by alkali stress, while their expression in young leaves decreased under alkali stress (Fig. 7). However, alkali stress did not influence the expression of *OsNRT2;1* in both tissues. Responses of *ammonium transporter* (*OsAMT*) *family* members to alkali stress were diverse. For example, alkali stress strongly stimulated the expression of *OsAMT1;3* in young leaves, while increased the expression of *OsAMT2;3* and *OsAMT3;2* in old leaves (Fig. 7).

## Discussion

### Growth

It is well-known that salt stress has stronger inhibition effect on the growth of old leaves than that of young leaves. This may be an adaptive strategy of plants to salt stress, and protects young organs via sacrificing old organs. We also had found this phenomenon in many extreme halophytes and glycophytes (unpublished data). In present study, we have observed that alkali stress has a stronger injury effect on old leaves than that of young leaves. Alkali stress strongly damaged the membrane system and photosynthetic pigment of old leaves even changed the ratio between different pigments (Fig. 1D).

### Ion Balance

Alkali stress showed a stronger effect on the ion balance in rice old leaves than young leaves. The results indicated that the effects of alkali stress on Na^+^, K^+^, Na^+^/K^+^ ratio, Cl^−^, SO_4_^2−^ and ammoniacal nitrogen in young leaves were no significant, but alkali stress strongly stimulated their accumulation in old leaves (Fig. 2). This may be an adaptive response of rice plants to alkali stress. Rice plants may protect young leaves from ion injury via a large accumulation of toxicant ions in old leaves. We also observed that, under alkali stress, both first and second leaves at bottom (old leaves) accumulated larger Na^+^ but other five leaves (one young leaf and four maturate leaves) all accumulated the low concentration of Na^+^ (unpublished data). Old leaf cells have larger vacuole, but young leaf cells only have dispersed miniature vacuoles. Rice plants can compartmentalize toxicant ions like Na^+^ into the larger vacuoles of old leaves to avoid the ion toxicity of whole green part (Fig. 2). Rice may have a specific regulatory mechanism of Na^+^ transmission into old leaves.

Na^+^ enters plant cells through the K^+^ transporter pathways and non-selective cation channels [31]. Under salt stress the *in vivo* Na^+^ metabolism of plants has least three processes: compartmentalization (at cellular and/or tissue levels), exclusion (from shoots into roots) and transportation (in vasculatures) of the ions. In *Arabidopsis*, the salt overly sensitive protein 1 (SOS1) functions in Na^+^ exclusion from root epidermal cells into the rhizosphere, which also may play a role in retrieving Na^+^ from leaf under severe salt stress [8]. The Ca^2+^-responsive AtSOS3–AtSOS2 (AtCIPK24-AtCBL4) protein kinase pathway mediates regulation of the expression and activities of Na^+^ transporters such as AtSOS1 and AtNHX, a Na^+^/H^+^ exchanger that mediates Na^+^ compartmentalization into vacuoles [31]. The rice SOS salt tolerance pathway has been identified and its functions have been shown as similar to that of the SOS pathway in *Arabidopsis* [32]. In *Arabidopsis* and some other plant species, the Na^+^/H^+^ exchanger (NHX) family has been shown to function in Na^+^ compartmentalization into vacuoles [8]. In rice leaves, SOS salt tolerance pathway and NHX family may play a role in retrieving Na^+^ from leaf cells to the vascular tissue and Na^+^ compartmentalization, separately [8]. Our results revealed that, under alkali stress, Na^+^ homeostasis of rice leaves might have a sophisticated regulating network. Under alkali stress, rice was able to change the ion distribution at whole plant level via altered expression of critical genes involved in ion balance. For example, the expression of several genes, such as *OsSOS1*, *Os CIPK24*, *OsNHX1*, *OsNHX2*, *OsHKT1;1*, *OsAKT1*, *OsHAK1*, *OsHAK7*, *OsHAK10*, *OsHAK16*, in old leaves was clearly stimulated by alkali stress, but their expression levels in young leaves were not affected (Figs. 4 and 5). Under alkali stress, the increased expression of *OsSOS1*, *OsCIPK24 and OsNHX* family might be helpful for Na^+^ compartmentalization and retrieving Na^+^ from old leaf cells to vascular tissue. Up-regulated expression of *OsHKT1;1*, *OsAKT1*, *OsHAK1*, *OsHAK7*, *OsHAK10* and *OsHAK16* might contribute to the larger accumulation of Na^+^ in old leaves of alkali stressed-rice. However, alkali stress did not increase the expression of *OsHKT1;3*, *OsHKT1;5*, and *OsHKT2;1* in rice old leaves (Fig. 4), suggesting that it was unlikely that the three genes contributed to the accumulation of Na^+^ in old leaves.

Ionic imbalance in plants is mainly caused by the influx of superfluous Na^+^ [6], [16]. Plants usually accumulate inorganic anions, such as Cl^−^, NO_3_^−^ and SO_4_^2−^, or synthesized organic anions to maintain ionic balance and pH homeostasis [16]. Our results revealed that, under alkali stress, young leaves did not accumulate Cl^−^, SO_4_^2−^, and organic acids, but old leaves accumulated the high concentrations of organic acids, Cl^−^ and SO_4_^2−^. This may be an adaptative response to Na^+^ excess in old leaves (Figs. 2 and 3), and plays important roles in the maintaining ionic balance and pH homeostasis in old leaves. In addition, we found that alkali stress stimulated the accumulation of soluble sugars in young leaves but did not increase its content in old leaves (Fig. 3E). This may be an adaptive response of rice to alkali stress, and soluble sugar possibly plays an important osmotic role in the young leaves of alkali-stressed rice.

### Nitrogen Nutrition

Plant survival and growth in saline environments is a result of adaptive processes such as ion transport and compartmentation, compatible solutes synthesis and accumulation. Many of these compatible solutes are N-containing compounds, such as amino acids and betaines, hence the nitrogen metabolism is of central importance for salt tolerance [33]. However, interference between salinity and nitrogen nutrition is a very complex network affecting almost all processes in plant metabolism and development. Our results indicated that alkali stress strongly influenced the nitrogen metabolism of rice leaves. Alkali stress reduced the NO_3_^−^ contents in both old and young leaves, but the reduction in young leaves was greater that in old leaves (Fig. 2E). Under alkali stress, the NO_3_^−^ content in old leaves was much higher than that in young leaves. The lacking of NO_3_^−^ in roots may be main reason for the deficiency of NO_3_^−^ in rice leaves under alkali stress. It has been reported that alkali stress limited NO_3_^−^ uptake of rice roots, and reduced NO_3_^−^ content of the roots [20], which may lead to the persistent lacking of NO_3_^−^ in stems and leaves. As the old leaf cells have larger vacuole (only miniature vacuoles for young leaf), these scarce NO_3_^−^ may be principally stored in rice old leaves to keep the NO_3_^−^ supply in stems and leaves. Rice may limit the transmission of NO_3_^−^ into young leaves. Accordingly, the two genes coding nitrate transporter, *OsNRT1;1* and *OsNRT1;2*, in old leaves were up-regulated by alkali stress, while their expression levels in young leaves were down-regulated (Fig. 7). This revealed that the two genes might contribute to the accumulation of NO_3_^−^ in the old leaves of alkali stressed-rice.

It was recognized that NO_3_^−^ is reduced to nitrite by nitrate reductase (NR) and then to NH_4_^+^ by nitrite reductase (NiR). NH_4_^+^ from both nitrate reduction and soil are incorporated into organic molecules by glutamine synthetase (GS) and glutamate synthase (Fd-GOGAT and NADH-GOGAT) or alternative glutamate dehydrogenase (GDH) pathway (reviewed by Shi et al. 2010) [21]. The expression of *OsFd-GOGAT* in rice leaves was more abundant than other *OsGOGAT* gene family members, and the expression of *OsGS2* also was more abundant than other *OsGS* gene family members (Fig. 6). Namely, *OsFd-GOGAT* and *OsGS2* are principally expressed in leaves and played crucial roles in the assimilation of NH_4_^+^ from photorespiration and other metabolic process. Compared both tissues, we found that the effect of alkali stress on the nitrogen metabolism of young leaves was stronger than that of old leaves. Alkali stress did not influence the expression of *OsFd-GOGAT* and *OsGS2* in old leaves, whereas mightily reduced their expression in young leaves. The decreased expression of *OsFd-GOGAT* and *OsGS2* in young leaves might be a response to NO_3_^−^ deficiency. The NO_3_^−^ deficiency in young leaves might cause the large reduction in *OsNR1* expression (Fig. 6A) and the subsequent lacking of free NH_4_^+^, which might be main reason why alkali stress sharply down-regulated the expression of *OsFd-GOGAT* and *OsGS2* in young leaves (Fig. 6). Moreover, the responses of the *OsAS*, *OsGDH* and *OsAMT* gene families in both tissues to alkali stress also showed that alkali stress has different effect on their nitrogen metabolism.

In summary, alkali stress only produced a small effect on the growth of young leaves, whereas strongly damaged the membrane system and photosynthetic pigment in old leaves. Old leaf cells have larger vacuole, but young leaf cells only have dispersed miniature vacuoles. Rice plants can compartmentalize toxicant ions, such as Na^+^ and Cl^−^, into the larger vacuoles of old leaves to avoid the ion toxicity of whole plant. Up-regulated expression of *OsHKT1;1*, *OsAKT1*, *OsHAK1*, *OsHAK7*, *OsHAK10* and *OsHAK16* might contribute to the larger accumulation of Na^+^ in old leaves of alkali stressed-rice. The effect of alkali stress on the nitrogen metabolism of young leaves was stronger than that of old leaves. Alkali stress reduced the NO_3_^−^ contents in both old and young leaves, but the reduction in young leaves was greater than that in old leaves. As old leaf cells have larger vacuole, these scarce NO_3_^−^ may be principally stored in rice old leaves. *OsNRT1;1* and *OsNRT1;2* might contribute to the accumulation of NO_3_^−^ in old leaves. NO_3_^−^ deficiency in young leaves might induce the large reduction in *OsNR1* expression and the subsequent lacking of free NH_4_^+^, which might be main reason why alkali stress sharply down-regulated the expression of *OsFd-GOGAT* and *OsGS2* in young leaves. Our results strongly indicated that, during adaptation of rice to alkali stress, young and old leaves have distinct mechanisms of ion balance and nitrogen metabolism regulation. However, most of research of salt stress and alkali stress still use the mixed sample of young and old tissues, which only provides the limited insights into stress tolerance mechanism. We propose that the comparative studies of young and old tissues may be important for abiotic stress tolerance research.

## Supporting Information

Table S1 Gene-specific primers used in real time PCR analysis.
(DOC)
