# Supplementary material for: Comparison of Ion Balance and Nitrogen Metabolism in Old and Young Leaves of Alkali-Stressed Rice Plants
Source: PLoS One. 2012 May 24;7(5):e37817. doi: 10.1371/journal.pone.0037817 (PMC3360002; doi:10.1371/journal.pone.0037817)
Supplement: Table S1 — Gene-specific primers used in real time PCR analysis. (DOC) [file pone.0037817.s001.doc]

| **Gene name** | **GenBank Accession No.** | **Forward primer (5’-3’)** | **Reverse primer (5’-3’)** | **Reference** |
| --- | --- | --- | --- | --- |
| *OsNHX1* | AB021878 | GTTCAAGAGTTACAACAAAGCACG | CAGCGGGAATACAAAAGCAG | [1] |
| *OsNHX2* | AY360145 | ACCAAGACGAAACACCCCTAC | AACCCAGCAACTACTCCAAGAA | [2] |
| *OsHKT1;1* | AJ491816 | ATTAGCAGAGCACTGTGGAGGAA | CCGACGAACCCGTAGGAAG | [3] |
| *OsHKT1;3* | *AJ491818* | *CAGTTCATCTACCAAAACAATCCA* | *AATACCTCACCACCAATCAGCA* | [3] |
| *OsHKT1;5* | DQ148410 | TGCCACCTTACACCACTTTCG | TGCCATACGCACTGATAACCTC | [3] |
| *OsHKT2;1* | AB061311 | GCATATTCACCCATTCTGGATTCAGT | CGATGGTGATGAGGCTGGAAAGT | [3] |
| *OsSOS1* | AY785147 | CTCCGTGCTCATAGAATCGC | ATACTCACTCAAGTGGGTCAATACC | [4] |
| *OsCBL4* | AK101368 | GGCATCGTTCGGATTTCAC | GAGATTCGCCTTTCTGCTGTT | [4] |
| *OsCIPK24* | AK102270 | AAGAAGCGGGTGGGGAGGT | GCGGTGGTTGAGGATGGTGT | [4] |
| *OsAKT1* | AY065970 | TACGACCGCCGATACAGAA | CCAAATAAGCCACAAAGAAGG | [5] |
| *OsHAK1* | AK119883 | TGGCGTTCCAGAGCGTG | GGGGATGATGATGAGGGTGTA | **[6]** |
| *OsHAK4* | AF129485 | CGTTCCCATCCGTCAGTAAA | CAGCCTCTGGTCTGGTTCGTC | **[6]** |
| *OsHAK7* | AJ427971 | GAACTCCAACTTCCTCAAGACG | AGATCATGCCGACTTCGACGAG | **[6]** |
| *OsHAK10* | AJ427972 | CGCTCTCGGCTGCTTTCCT | TAACCGCCAATCCTGACGC | **[6]** |
| *OsHAK16* | AJ427973 | AGCGACTGTGTGCTAAACCC | CATAGATGCCAATCCCTGAGA | **[6]** |
| *OsNR1* | AK121810 | CCTACTACTAAATTATACGCACCG | CAGGAAGGAATCAACCGCTA | [7] |
| *OsNiR* | AK103604 | CAAATCAGTGTTCCGATAGGTAA | GGCTGGAGACGGTGGTG | [8] |
| *OsGS1;1* | AB037595 | CCGTCTGTCGGCATTTCTG | GGGATGGGCTTGGGGTC | [9] |
| *OsGS1;2* | AB180688 | GCCCACAGGGACCATACTACT | CGTTGATGCCACTGATGTTGAT | [9] |
| *OsGS1;3* | AB180689 | CCGATTCCGACGAACAACC | GCTCCCGCCGCACAGT | [9] |
| *OsGS2* | X14246 | AGTATGCGTGAAGATGGAGGAT | GCCCCACCCGAATAGAGC | [9] |
| *OsNADH-GOGAT1* | AB008845 | TTGCGGTTACAAGACACTCTACTG | GCTCCCGTCCCTCCATCA | [9] |
| *OsNADH-GOGAT2* | AB274818 | CCTGTCGAAGGATCGTGAAGGTCAAACC | TGCATGGCCCTACTGTCTTCGCATCA | [9] |
| *OsFd-GOGAT* | AJ132280 | TGGTCTCCGCCCAGCAC | CAGTTTGTAGGTCAACCGTTATCAT | [9] |
| *OsGDH1* | AK071839 | TTCTTCCTTCCCACTACCAAAC | TCCCAAGCAGCGAGCC | [9] |
| *OsGDH2* | AB189166 | GGCCATTAACAACACTCATA | ACGCCGATCTATCTTGAAT | [9] |
| *OsGDH3* | AB035927 | TATGCTACTGAGGCTTTATTGACTG | GCCACCTTTCTGATGGATGA | [9] |
| *OsAS* | D83378 | GCCCTATTTACCTAAGCACATTC | AGGCTGCGTCCCATTCA | [10] |
| *OsNRT1;1* | AF140606 | GGGCAGAGTTCAGCAATCG | GGAAGGACGCCGCAGGT | [11] |
| *OsNRT1;2* | AY305030 | GCGGCGAGTCCCTGAG | CGACGGCGTAGATGAATGA | [12] |
| *OsNRT2;1* | AK072215 | ACGGCACAAAGTACAAGACG | CCACTGCGGGAAGTAGATG | [13] |
| *OsAMT1;1* | AK073718 | GCCTCCAACAGCAACAACC | CCAAACAGAAACTGGCAATCA | [14] |
| *OsAMT1;2* | NM_001053990 | CACGGTGGCGATGAAAGG | TTGGAGATGGTGGTGAAGGAC | [14] |
| *OsAMT1;3* | AK107204 | TCAAGCAGGTCCCACAGG | TGAGGAAGGCGGAGTAGATG | [14] |
| *OsAMT2;1* | AB051864 | GATGAATCACGCCGAAACAC | GCACGGACGAATCGCTACTT | [14] |
| *OsAMT2;2* | AB083582 | CGACCAAGGACAGGGAGA | CACGGCGAGCGAGGAG | [14] |
| *OsAMT2;3* | AK102106 | GTTCACCCCGCTCTGGC | CCGCTCCCTGTCGCTCTT | [14] |
| *OsAMT3;1* | AK120352 | CCAACTGCTGAAAAGTGAAAACG | TGCTTCGCATACGGCTGAC | [14] |
| *OsAMT3;2* | AK069311 | CCCAGTTCGGCAAGCAG | TGGCGAGGCAGATGAGG | [14] |
| *OsAMT3;3* | AK108711 | GAGATTCCCGCCCAACAA | TCCACCCAAGCCACAGC | [14] |
| *OsUBQ5* | AK061988 | ACCACTTCGACCGCCACTACT | ACGCCTAAGCCTGCTGGTT |  |

NR, nitrate reductase; NiR, nitrite reductase；GOGAT, glutamate synthase; GS, glutamine synthetase; GDH, glutamate dehydrogenase; AS, asparagine synthetase. NRT, nitrate transporter; AMT, ammonium transporter; NHX, Na^+^/H^+^ exchanger; HKT, high affinity K^+^ transporter; HAK, KUP/HAK/KT K^+^ transporter; AKT, low affinity K^+^ transporter; SOS, salt overly sensitive.

**References**

1. Fukuda A, Nakamura A, Tagiri A, Tanaka H, Miyao A, et al. (2004) Function, intracellular localization and the importance in salt tolerance of a vacuolar Na^+^/H^+^ antiporter from rice. Plant Cell Physiol 45: 146-159.

2. Negrão S, Courtois B, Ahmadi N, Abreu I, Saibo N, et al. (2011) Recent updates on salinity stress in rice: from physiological to molecular responses. Crit Rev Plant Sci 30: 329-377.

3. Platten JD, Cotsaftis O, Berthomieu P, Bohnert H, Davenport RJ, et al. (2006) Nomenclature for HKT transporters, key determinants of plant salinity tolerance. Trends Plant Sci 11: 372.

4. Martínez-Atienza J, Jiang X, Garciadeblas B, Mendoza I, Zhu JK, et al. (2007) Conservation of the salt overly sensitive pathway in rice. Plant Physiol 143: 1001-1012.

5. Fuchs I, St lzle S, Ivashikina N, Hedrich R (2005) Rice K^+^ uptake channel OsAKT1 is sensitive to salt stress. Planta 221: 212-221.

6. Bañuelos MA, Garciadeblas B, Cubero B, Rodríguez-Navarro A (2002) Inventory and functional characterization of the HAK potassium transporters of rice. Plant Physiol 130: 784-795.

7. Choi HK, Kleinhofs A, An G (1989) Nucleotide sequence of rice nitrate reductase genes. Plant Mol Biol 13: 731-733.

8. Terada Y, Aoki H, Tanaka T, Morikawa H, Ida S (1995) Cloning and nucleotide sequence of a leaf ferredoxin-nitrite reductase cDNA of rice. Biosci Biotech Bioch 59: 2183-2185.

9. Kusano M, Tabuchi M, Fukushima A, Funayama K, Diaz C, et al. (2011) Metabolomics data reveal a crucial role of cytosolic glutamine synthetase 1; 1 in coordinating metabolic balance in rice. Plant J 66: 456-466.

10. Sasaki T, Song J, Koga-Ban Y, Matsui E, Fang F, et al. (1994) Toward cataloguing all rice genes: large-scale sequencing of randomly chosen rice cDNAs from a callus cDNA library. Plant J 6: 615-624.

11. Lin CM, Koh S, Stacey G, Yu SM, Lin TY, et al. (2000) Cloning and functional characterization of a constitutively expressed nitrate transporter gene, *OsNRT1*, from rice. Plant Physiol 122: 379-388.

12. Tanaka T, Antonio BA, Kikuchi S, Matsumoto T, Nagamura Y, et al. (2008) The rice annotation project database (RAP-DB): 2008 update. Nucleic Acids Res 36: D1028.

13. Cai C, Wang JY, Zhu YG, Shen QR, Li B, et al. (2008) Gene structure and expression of the high-afﬁnity nitrate transport system in rice roots. J Integr Plant Biol 50: 443-451.

14. Li BZ, Merrick M, Li SM, Li HY, Zhu SW, et al. (2009) Molecular basis and regulation of ammonium transporter in rice. Rice Sci 16: 314-322.
